# Prevalence and risk factors of portal vein thrombosis following hepatectomy: a systematic review and meta‑analysis

**DOI:** 10.20452/wiitm.2025.17929

**Published:** 2025-01-15

**Authors:** Yang Qun, Feng Meiying, Yao Weiming, He Dan

**Affiliations:** Department of General Surgery, Jingdezhen Hospital of Traditional Chinese Medicine, Jingdezhen, Jiangxi, China; Department of General Surgery and Clinical Nutrition, Jingdezhen Hospital of Traditional Chinese Medicine, Jingdezhen, Jiangxi, China; Department of Gynecology and Obstetrics, Jingdezhen Maternity and Child Care Hospital, Jingdezhen, Jiangxi, China

**Keywords:** hepatectomy, portal vein thrombosis, risk factors, systematic review

## Abstract

**INTRODUCTION:**

The prevalence and risk factors of portal vein thrombosis (PVT) are largely unclear, with an increasing number of studies reporting inconsistent results.

**AIM:**

The current study aimed to evaluate the prevalence and risk factors of PVT following hepatectomy through a systematic review and meta‑analysis.

**MATERIALS AND METHODS:**

A comprehensive literature search was conducted across multiple databases (PubMed, Embase, and the Cochrane Library) to identify relevant studies. Prospective and retrospective studies reporting on PVT following hepatectomy were included. The Newcastle‑Ottawa Scale (NOS) was used to assess study quality, and the random effects model was used to analyze the prevalence and risk factors.

**Result:**

A total of 15 studies involving 5145 patients were included in the current meta‑analysis. The pooled prevalence of PVT following hepatectomy was 9% (95% CI, 7%–12%) with substantial heterogeneity (I2 = 93.1%). Subgroup analyses showed that a prospective design and larger sample size were associated with lower prevalence rates. PVT prevalence was higher among the patients undergoing simultaneous splenectomy and hepatectomy. Liver cirrhosis (odds ratio [OR], 5.18; 95% CI, 1.85–14.47), portal vein resection (OR, 5.07; 95% CI, 2.2–11.66), and right‑sided hepatectomy (OR, 6.26; 95% CI, 1.8–21.76) were significant risk factors for PVT.

**CONCLUSIONS:**

PVT is a notable complication following hepatectomy, with an overall prevalence of 9%. Specific factors that significantly increase the risk of PVT include liver cirrhosis, portal vein resection, and right‑sided hepatectomy.

## INTRODUCTION

Portal vein thrombosis (PVT) is a significant yet often underrecognized complication of hepatectomy, with substantial clinical implications for patient outcomes.^1^^,^^2^ Hepatectomy is a cornerstone treatment for various liver conditions, including hepatocellular carcinoma and metastatic liver tumors.^3^ Despite the proven efficacy of hepatectomy in treating these conditions, potential complications, such as PVT, pose serious challenges.^4^^,^^5^ PVT can lead to compromised liver function, impaired regeneration, prolonged hospital stay, and increased postoperative mortality.^1^ Thus, there is a critical need for a deeper understanding of its prevalence and risk factors to guide clinical practice and improve patient care.

The reported incidence of PVT after hepatectomy varies considerably. This variation is largely attributed to differences in patient characteristics, surgical techniques, and study methodologies. Specific subgroups of patients, such as those undergoing extended hepatectomy or those with underlying liver cirrhosis, are at a particularly high risk of developing PVT.^4^^,^^6^^,^^7^ For instance, individuals with liver cirrhosis experience altered portal hemodynamics and hypercoagulability, which significantly increase their susceptibility to PVT.^8^^,^^9^ These factors complicate perioperative management and underscore the need for a comprehensive evaluation of the PVT incidence and its determinants.^10^ A standardized approach to assessing and reporting PVT incidence could contribute to a clearer understanding of this complication across different patient populations.

**TABLE 1:** Baseline characteristics of the included studies

Study	Design	Country	Enrollment period	Age, y (SD or Range)	Sample size, n	Diagnosis	PVT detection method	Type of hepatectomy	Centers involved
Qi et al^5^	Retrospective	China	2018–2023	58 (11.6)	1029	NA	NA	Major (right/left)	Single-center
Katano et al^9^	Retrospective	Japan	2004–2021	68.5 (34–84)	102	NA	Contrast-enhanced CT	Hepatectomy and/or splenectomy	Single-center
Lemaire^14^	Retrospective	France	2012–2019	68 (48–76)	86	Perihilar cholangiocarcinoma	Doppler ultrasound or CT	Major hepatectomy	Single-center
Terasaki et al^34^	Retrospective	Japan	2009–2020	70 (35–83)	247	NA	Contrast-enhanced CT	Right hepatectomy, including cases with and without PVR	Single-center
Takata et al^18^	Retrospective	Japan	2014–2019	73 (38–93)	65	Hepatocellular carcinoma	Contrast-enhanced CT	Major or minor hepatectomy	Single-center
Okuno et al^33^	Retrospective	Japan	2015–2018	68 (20–87)	295	NA	Contrast-enhanced CT	Right-sided hepatectomy	Single-center
Cao et al^28^	Retrospective	Japan	2002–2018	68 (32–88)	177	Perihilar cholangiocarcinoma	Doppler ultrasound or CT	Major hepatectomy	Single-center
Onda et al^17^	Retrospective	Japan	2009–2019	63.6 (13.3)	398	Benign or malignant liver disease	Contrast-enhanced CT	Anatomical and partial hepatectomy	Single-center
Mori et al^32^	Retrospective	Japan	2006–2016	68.9 (9.9)	622	Primary liver cancer	Doppler ultrasound or CT	Laparoscopic and open hepatectomy	Single-center
Uchida et al^35^	Retrospective	Japan	2009–2016	72 (37–87)	81	Perihilar cholangiocarcinoma	Contrast-enhanced CT	Major hepatectomy with caudate lobectomy	Single-center
Han et al^29^	Retrospective	Korea	2009–2014	57.4 (13.1)	534	NA	NA	Major and minor hepatectomy	Single-center
Matsui et al^31^	Prospective	Japan	2012–2014	NA	81	NA	Contrast-enhanced CT	Hepatectomy and/or splenectomy	Single-center
Blasi et al^27^	Prospective	Spain	2011–2014	64 (54–72)	27	Cholangiocarcinoma	NA	Major hepatectomy	Single-center
Kuboki et al^30^	Retrospective	Japan	2000–2013	NA	1193	NA	Doppler ultrasound or CT	Major and minor hepatectomy	Single-center
Yoshiya et al^36^	Retrospective	Japan	2009–2012	66.7 (0.8)	208	Primary or metastatic liver tumors	Contrast-enhanced CT	Major hepatectomy	Single-center

Abbreviations: CT, computed tomography; NA, not available; PVR, portal vein resection; PVT, portal vein thrombosis

Several risk factors for PVT following hepatectomy have been proposed, with liver cirrhosis consistently emerging as a major predictor due to its impact on hepatic vasculature and the overall coagulation profile.^11^ Patients with cirrhosis may develop a hypercoagulable state caused by imbalanced pro‑ and anticoagulant factors, as well as endothelial dysfunction, all of which increase the risk of thrombosis.^12^^,^^13^ Apart from liver cirrhosis, intraoperative factors, such as blood transfusion, prolonged operative time, and specific surgical techniques (eg, portal vein resection [PVR]) have been linked to an increased PVT risk.^4^^,^^5^^,^^14^ Blood transfusions may contribute to a proinflammatory and hypercoagulable state, thereby exacerbating the risk of PVT. The invasive nature of PVR, which often involves extensive vascular manipulations, is associated with endothelial damage, a key factor predisposing patients to thrombus formation.^14^^,^^15^^,^^16^ Prolonged use of the Pringle maneuver, during which blood flow to the liver is temporarily occluded, can also exacerbate ischemia‑reperfusion injury, further increasing the likelihood of PVT.^17^^,^^18^ Understanding these risk factors is crucial for informing perioperative decision‑making and improving patient outcomes. Mitigating the risk of PVT requires targeted perioperative interventions.^1^ Perioperative anticoagulation is an important consideration for high‑risk patients, such as those with liver cirrhosis or those undergoing extensive vascular procedures.^19^^,^^20^ However, anticoagulation in these patients must be carefully balanced against the concurrent risk of bleeding.^20^^,^^21^ Proactive identification of modifiable risk factors and tailoring perioperative care to individual patient needs are essential for minimizing the incidence of PVT, reducing postoperative morbidity, and improving overall outcomes.

**TABLE 2:** Quality evaluation of the included studies based on the Newcastle‑Ottawa Scale criteria

Study	a	b	c	d	e	f	g	h	i	Score	Overall quality
Qi et al^5^	1	1	1	1	1	0	1	1	1	8	High
Katano et al^4^	1	1	1	1	0	0	1	1	0	6	Moderate
Lemaire et al^14^	1	1	1	1	0	0	1	1	0	6	Moderate
Terasaki et al^34^	1	1	1	1	0	0	1	1	0	6	Moderate
Takata et al^18^	1	1	1	1	0	0	1	1	0	6	Moderate
Okuno et al^33^	1	1	1	1	0	0	1	1	0	6	Moderate
Cao et al^28^	1	1	1	1	1	0	1	1	0	7	High
Onda et al^17^	1	1	1	1	1	0	1	1	1	8	High
Mori et al^32^	1	1	1	1	0	0	1	1	0	6	Moderate
Uchida et al^35^	1	1	1	1	0	0	1	1	0	6	Moderate
Han et al^29^	1	1	1	1	0	0	1	1	0	6	Moderate
Matsui et al^31^	1	1	1	1	0	0	1	1	0	6	Moderate
Blasi et al^27^	1	1	1	1	0	0	1	1	0	6	Moderate
Kuboki et al^30^	1	1	1	1	1	0	1	1	1	8	High
Yoshiya et al^36^	1	1	1	1	1	0	1	1	0	7	High

1. Representativeness of the exposed cohort

2. Selection methods for the nonexposed cohorts

3. Ascertainment of exposure

4. Outcome of interest not present at the start of the study

5. Study controlled for confounders

6. Study controlled for additional factors

7. Assessment of exposure

8. Follow‑up period long enough for outcomes to occur

Scores: 0–3, low quality; 4–6, moderate quality; 7–9 high quality

9. Adequacy of follow‑up of cohorts

## AIM

The objective of this systematic review and meta‑analysis was to estimate the pooled prevalence of PVT following hepatectomy and to identify key risk factors validated through multivariable or adjusted analyses. By consolidating the available evidence, we aimed to provide actionable insights into the prevention and management of PVT, ultimately contributing to improved clinical outcomes of patients undergoing hepatectomy.

## MATERIALS AND METHODS

Study design This systematic review and meta‑analysis adhered to the Preferred Reporting Items for Systematic Reviews and Meta‑Analyses guidelines.^22^ The primary objective was to estimate the prevalence of PVT following hepatectomy and to identify the risk factors for its occurrence.

### Search strategy

A comprehensive literature search was performed across multiple databases (PubMed, Embase, and the Cochrane Library) to identify relevant studies published since the database inception until August 7, 2024, without language restrictions. The search strategy used a combination of Medical Subject Headings terms and relevant key words, *such as portal vein thrombosis, hepatectomy, liver surgery, prevalence, and risk factors*, and their variants. Reference lists of the retrieved articles were manually screened to identify any additional studies that met the inclusion criteria.

### Eligibility criteria

Studies were eligible for inclusion if they met the following criteria: 1) reported quantitative data on the prevalence of PVT or included a multivariable or adjusted analysis of risk factors associated with PVT following hepatectomy; 2) involved adult patients undergoing hepatectomy for any indication; 3) were original research articles, including observational studies (eg, cohort, cross‑sectional, and case‑control studies).

Studies were excluded if they were case reports, letters, conference abstracts, reviews, or animal studies, or if they lacked sufficient data on PVT prevalence or associated risk factors.

### Data extraction and management

Two independent reviewers (FM and YW) extracted data using a standardized data extraction form. The extracted data included study characteristics (author, year of publication, country), patient demographics (age, sex, presence of liver cirrhosis), surgical details (type of hepatectomy, PVR, Pringle maneuver duration), and outcomes (prevalence of PVT, identified risk factors, and effect estimates). Any disagreements between the reviewers were resolved through discussion or by consulting a third reviewer (HD). The EndNote soft‑ ware (Clarivate, Philadelphia, Pennsylvania, United States) was used for reference management.

**FIGURE 1:**
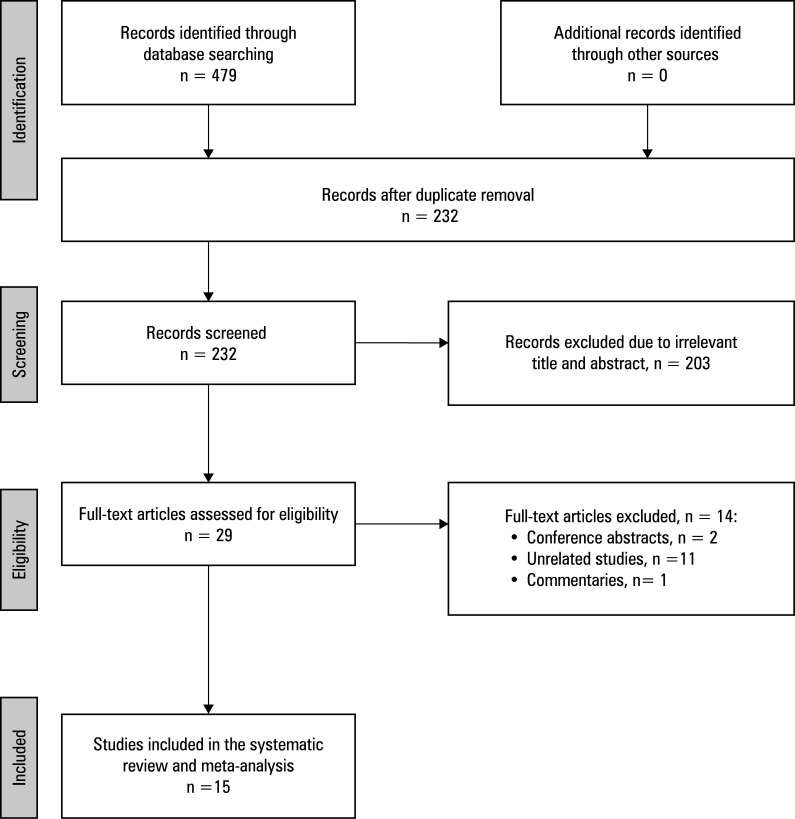
PRISMA flow diagram of the study selection process

### Quality assessment

The quality of the included papers was assessed using the Newcastle‑Ottawa Scale (NOS) for observational studies.^23^ The NOS evaluates study quality across 3 domains: selection of study groups, comparability of groups, and assessment of outcomes.23 Each study was as‑ signed a score of up to 9 points, with higher scores indicating better quality. We assigned scores of 0 to 3, 4 to 6, and 7 to 9 for low, moderate, and high quality of studies, respectively.

### Statistical analysis

This meta‑analysis was performed to evaluate the pooled prevalence (primary outcome) and risk factors (secondary outcomes) of PVT following hepatectomy. Event rates and their corresponding 95% CIs were extracted or calculated from the included studies for prevalence estimation. To stabilize variance and normalize the distribution, log‑transformed prevalence rates and SEs were used. The pooled prevalence was calculated using the DerSimonian–Laird random effects model to account for the expected heterogeneity across studies. Heterogeneity was assessed using the Cochrane Q test and quantified by the I^2^ statistic, with I^2^ greater than 50% indicating substantial heterogeneity.^24^ Subgroup analyses were conducted based on study design, sample size, study quality, type of surgery, and geographic region to explore potential sources of heterogeneity. The leave‑one‑out sensitivity analyses were performed by sequentially excluding individual studies to evaluate the robustness of the overall results. We also performed sensitivity analyses through excluding low‑quality studies (based on the risk of bias assessment) to evaluate their impact on the overall pooled effect estimate. For the risk factors, odds ratios (ORs) with their 95% CIs were extracted, and log‑transformed ORs and SEs were computed for each study. The random effects model was used for all meta‑analyses due to the substantial heterogeneity observed among the included studies. Publication bias was evaluated using funnel plots and the Egger or Begg regression test, with a P value below 0.05 indicating significant publication bias.^25^ If a bias was detected, the trim‑and‑fill method was applied to adjust the pooled effect size.^26^ All statistical analyses were performed using the Stata software, version 12.0 (StataCorp, College Station, Texas, United States).

### Ethical approval

This study did not require approval of a bioethics committee.

## RESULTS

Study selection A comprehensive literature search yielded 479 records. After removal of duplicates, the remaining 232 records were screened based on the title and abstract, leading to the exclusion of 203 articles. Subsequently, 29 full‑text articles were assessed for eligibility, and 14 were excluded for the following rea‑ sons: conference abstracts (n = 2), unrelated studies (n = 11), and commentaries (n = 1). Ultimately, 15 studies met the inclusion criteria and were included in the systematic review and meta‑analysis^4^^,^^5^^,^^14^^,^^17^^,^^18^^,^^27^^,^^28^^,^^29^^,^^30^^,^^31^^,^^32^^,^^33^^,^^34^^,^^35^^,^^36^ FIGURE 1 .

**TABLE 3:** Subgroup analysis of the prevalence of portal vein thrombosis following hepatectomy

Outcomes	Number of studies	OR (95% CI)	Heterogeneity, *I2*, %
Pooled results	15	0.09 (0.07–0.12)	93.1
Study design			
Prospective studies	2	0.14 (0.06–0.22)	0
Retrospective studies	13	0.09 (0.07–0.11)	93.9
Sample size			
More than 500	4	0.026 (0.019–0.033)	29.9
Less than 500	9	0.014 (0.09– 0.19)	90.4
Study quality (NOS score)			
High (≥8 points)	3	0.05 (0.02–0.09)	95.7
Low / moderate (<8 points)	12	0.11 (0.08–0.15)	91.6
Type of surgery			
Hepatectomy	13	0.08 (0.06–0.1)	92.9
Simultaneous splenectomy and hepatectomy	2	0.21 (0.06–0.36)	81.2
Study region			
Asia	13	0.09 (0.07–0.12)	91.57
Europe	2	0.09 (0.04–0.14)	0

Abbreviations: NOS, Newcastle‑Ottawa Scale; OR, odds ratio

**TABLE 4:** A leave‑one‑out sensitivity analysis involving sequential exclusion of individual studies

Excluded study	Estimate	95% CI
Qi et al^5^	0.105	0.075–0.134
Katano et al^4^	0.083	0.06–0.106
Lemaire et al^14^	0.094	0.069–0.118
Terasaki et al^34^	0.074	0.0536–0.093
Takata et al^18^	0.088	0.0645–0.112
Okuno et al^33^	0.096	0.0708–0.121
Cao et al^28^	0.099	0.0736–0.125
Onda et al^17^	0.086	0.063–0.109
Mori et al^32^	0.101	0.0742–0.129
Uchida et al^35^	0.092	0.067–0.116
Han et al^29^	0.101	0.074–0.127
Matsui et al^31^	0.091	0.0672–0.116
Blasi et al^27^	0.092	0.068–0.116
Kuboki et al^30^	0.105	0.0755–0.134
Yoshiya et al^36^	0.093	0.068–0.118

### Characteristics of the included studies

General characteristics of the included studies are summarized in TABLE 1. The studies were conducted across 5 countries, with a majority having been carried out in Japan (n = 11). The remaining 4 studies were conducted in China, Korea, France, and Spain. Sample sizes ranged from 27 to 1193 patients, with study enrollment periods spanning from 2000 to 2023. Most of the included studies were retrospective (n = 13), while 2 were prospective.27, 31 The primary methods of PVT detection were contrast‑enhanced computed tomography and Doppler ultrasound.

The quality of the included studies was assessed using the NOS criteria TABLE 2. Overall quality scores ranged from 6 to 8, with 5 studies rated to be of high quality,^5^^,^^17^^,^^28^^,^^30^^,^^36^ and the remaining ones rated to be of moderate quality.^4^^,^^14^^,^^18^^,^^27^^,^^29^^,^^31^^,^^32^^,^^33^^,^^34^^,^^35^ Most studies demonstrated good representative‑ ness of the cohort and appropriate selection of controls; however, limitations in controlling for confounders and the adequacy of follow‑up were noted, potentially affecting the robustness of the findings.

### Pooled prevalence of portal vein thrombosis

**FIGURE 2:**
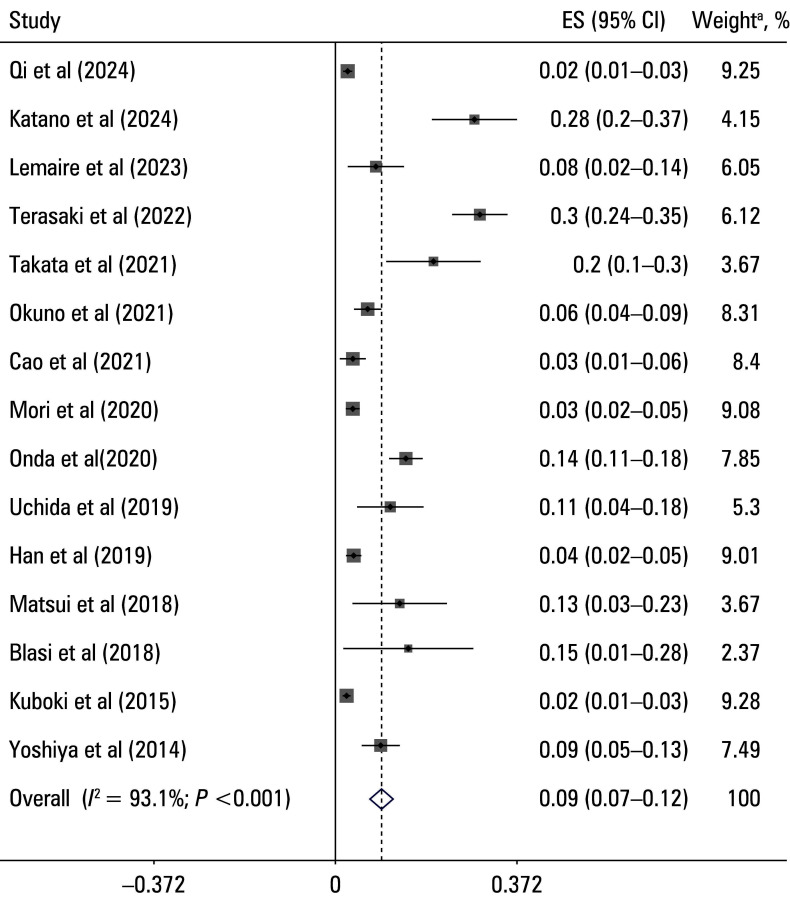
Forest plot of the prevalence of portal vein thrombosis following hepatectomy

The overall pooled prevalence of PVT following hepatectomy was 9% (95% CI, 7%–12%), as depicted in FIGURE 2. Substantial heterogeneity was observed among the included studies (I2 = 93.1%). The prevalence estimates in individual studies ranged from 2% to 30%, reflecting considerable variability in the reported rates of PVT.

To explore potential sources of heterogeneity, subgroup analyses were conducted based on study design, sample size, study quality, type of surgery, and study region TABLE 3. The pooled prevalence of PVT was higher in the prospective studies (OR, 0.14; 95% CI, 0.06–0.22), as compared with the retrospective ones (OR, 0.09; 95% CI, 0.07–0.11). Notably, no heterogeneity was observed among the prospective studies (I^2^ = 0%), whereas substantial heterogeneity was noted among the retrospective ones (I^2^ = 93.9%). The studies with a sample sizes greater than 500 reported a lower prevalence of PVT (OR, 0.026; 95% CI, 0.019–0.033; I^2^ = 29.9%) than those with smaller sample sizes (OR, 0.014; 95% CI, 0.09–0.19; I^2^ = 90.4%). The studies with NOS scores of 8 or more demonstrated a lower prevalence of PVT (OR, 0.05; 95% CI, 0.02–0.09), as compared with those with lower scores (OR, 0.11; 95% CI, 0.08–0.15), with high heterogeneity observed in both subgroups, particularly in the high‑quality group (I^2^ = 95.7%). The prevalence of PVT was also higher in the patients undergoing simultaneous splenectomy and hepatectomy (OR, 0.21; 95% CI, 0.06–0.36; I^2^ = 81.2%), as compared with those undergoing hepatectomy alone (OR, 0.08; 95% CI, 0.06–0.1). The studies conducted in Asia reported a pooled PVT prevalence of 9% (95% CI, 7%–12%; I^2^ = 91.57%), while the European studies reported a similar prevalence (OR, 0.09; 95% CI, 0.04–0.14) but with no heterogeneity (I^2^ = 0%).

**TABLE 5:** Risk factors for portal vein thrombosis following hepatectomy

Risk factor	Number of studies	Pooled OR (95% CI)	Heterogeneity, I2, %
Liver cirrhosis	2	5.177 (1.853–14.47)	0
Operative time	4	1.001 (0.995–1.007)	29.78
Blood loss	2	1.003 (0.902–1.115)	0
Portal vein resection	2	5.07 (2.204–11.661)	30.93
Right-sided hepatectomy	3	6.259 (1.8–21.761)	61.69
Pringle maneuver use	2	1.65 (0.759–3.589)	75.28
Postoperative PV angle	2	15.63 (3.22–75.88)	0
Remnant PV diameter	2	4.6 (0.39–54.73)	83.49

Abbreviations: PV, portal vein; others, see TABLE 3

a Weights are calculated with the random effects analysis.

Abbreviations: ES, estimate

### Sensitivity analysis and publication bias

**FIGURE 3:**
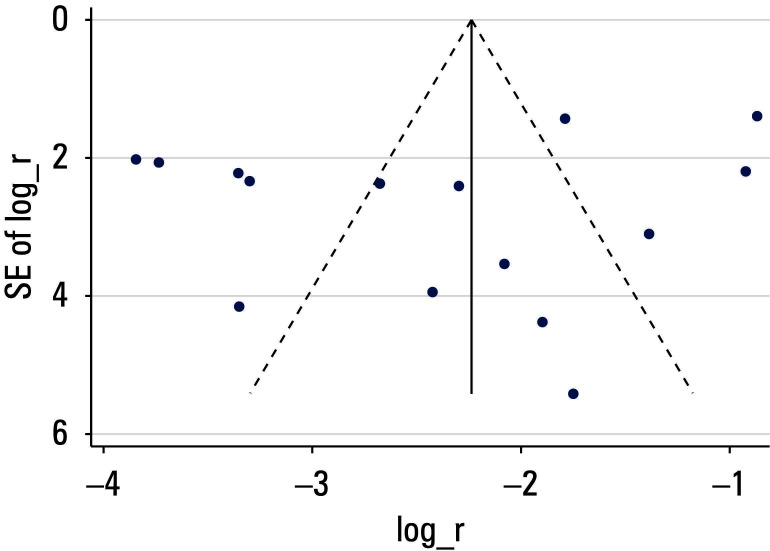
Funnel plot for assessing publication bias

**FIGURE 4:**
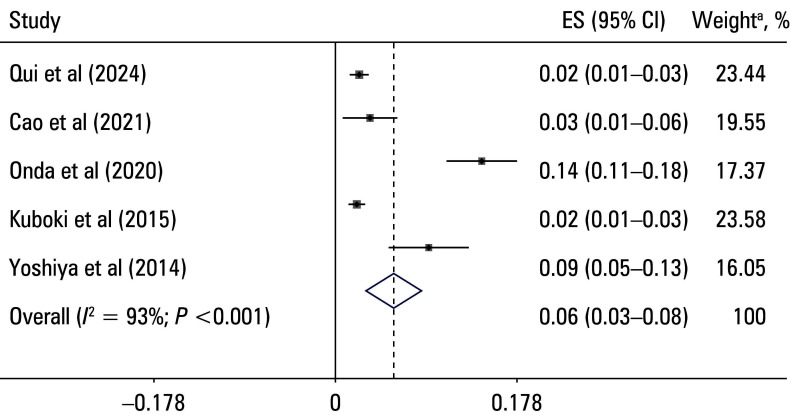
Forest plot of the prevalence of portal vein thrombosis following hepatectomy after removing lower‑quality studies

A leave one‑out sensitivity analysis was performed to evaluate the robustness of the pooled prevalence estimate TABLE 4. The results indicated that no single study significantly influenced the overall estimate, which remained stable at approximately 9% to 12%, suggesting robustness of the findings. We also performed sensitivity analyses involving exclusion of lower‑quality studies to evaluate their impact on the overall pooled effect, and the result showed that the pooled prevalence of PVT following hepatectomy was 6% (95% CI, 3%–8%) in the included studies of high quality FIGURE 3. To assess the potential publication bias, a funnel plot was constructed FIGURE 4. Visual inspection suggested some potential asymmetry, but statistical analysis (Egger test: P = 0.62) indicated no significant evidence of a publication bias.

### Risk factors for portal vein thrombosis

The pooled ORs for various risk factors associated with PVT are presented in TABLE 5. Liver cirrhosis was significantly associated with an increased risk of PVT (OR, 5.18; 95% CI, 1.85–14.47; I^2^ = 0%). The patients undergoing PVR also had an elevated risk of PVT (OR, 5.07; 95% CI, 2.2–11.66; I^2^ = 30.93%). Right‑sided hepatectomy was significantly associated with a higher risk of PVT (OR, 6.26; 95% CI, 1.8–21.76; I^2^ = 61.69%). Moreover, a wider post‑ operative portal vein angle was strongly associated with an increased PVT risk (OR, 15.63; 95% CI, 3.22–75.88; I^2^ = 0%).

Other factors, such as operative time (OR, 1.001; 95% CI, 0.995–1.007; I^2^ = 29.78%), blood loss (OR, 1.003; 95% CI, 0.902–1.115; I^2^ = 0%), Pringle maneuver use (OR, 1.65; 95% CI, 0.76–3.59; I^2^ = 75.28%), and remnant portal vein diameter (OR, 4.6; 95% CI, 0.39–54.73; I^2^ = 83.49%) did not show significant associations with the PVT risk.

### DISCUSSION 

This meta‑analysis provides a comprehensive evaluation of the prevalence and risk factors of PVT following hepatectomy. We showed that despite relatively low prevalence (9%), PVT is a significant postoperative complication with substantial implications for patient outcomes. The most critical risk factors identified were liver cirrhosis, PVR, right‑sided hepatectomy, and widened postoperative portal vein angle. These findings emphasize the need for careful patient selection, perioperative planning, and close post‑ operative monitoring in high‑risk populations.

The pooled prevalence of PVT following hepatectomy was estimated at 9%, with substantial heterogeneity. The variation in prevalence, ranging from 2% to 30%, may reflect differences in study design, surgical expertise, and patient characteristics, highlighting the need for standardized diagnostic criteria and protocols. Our finding that liver cirrhosis significantly increased PVT risk, which is consistent with earlier reports, underscores the association between hypercoagulable state and cirrhosis mediated by portal hypertension, impaired liver function, and endothelial dysfunction.^37^^,^^38^ Similarly, the high risk associated with PVR is in line with prior studies indicating that extensive vascular manipulation elevates the thrombotic risk due to endothelial injury.^15^^,^^16^ These results emphasize the importance of vigilant postoperative management in these high‑risk groups. The identified risk factors for PVT, including liver cirrhosis, PVR, right‑sided hepatectomy, and widened postoperative portal vein angle, reflect the clinically relevant underlying biological mechanisms. Cirrhosis contributes to hypercoagulable state, highlighting the need for enhanced monitoring and potential low‑dose anticoagulation to prevent PVT, despite the inherent bleeding risk.^20^^,^^39^^,^^40^ The increased PVT risk with PVR and right‑sided hepatectomy likely results from vascular disruption and altered portal flow dynamics. Minimizing endothelial injury and maintaining optimal venous flow are essential strategies to reduce the PVT risk.^1^ The strong association between a widened portal vein angle and PVT suggests that anatomical alterations may lead to venous stasis, necessitating surgical approaches that preserve optimal portal vein alignment. These findings support tailored perioperative interventions, such as anticoagulation and careful monitoring in high‑risk patients. Further studies should focus on optimizing surgical techniques and evaluating prophylactic measures to reduce the incidence of PVT and improve patient outcomes.

This meta‑analysis has several strengths, including a large pooled sample size, robust statistical methodologies, and a comprehensive assessment of both patient‑related and procedural risk factors for PVT. These strengths enhance the generalizability of our findings and provide valuable insights for both clinicians and researchers seeking to mitigate the PVT risk after hepatectomy.

a Weights are calculated with the random effects analysis.

Abbreviations: see FIGURE 2

However, certain limitations must be acknowledged. Firstly, we observed substantial heterogeneity across the included studies. Heterogeneity is a common challenge in systematic reviews, particularly when studies vary in clinical settings, patient populations, and diagnostic criteria. To explore potential sources of heterogeneity, we conducted subgroup analyses based on various factors. However, we were unable to identify any single factor that fully accounted for the observed heterogeneity. Also, the leave‑one‑out sensitivity analysis showed that omitting any individual study did not significantly affect the overall pooled effect. Understandably, these analyses suggest that heterogeneity is likely multifactorial and cannot be easily resolved through subgroup or sensitivity analyses alone. Consistently, sensitivity analyses involving exclusion of lower‑quality studies did not substantially alter the effect estimate. Consequently, despite the inherent variability among studies, the consistency of our findings across multiple analyses supports the robustness and reliability of the overall pooled effect estimate. Additionally, while a publication bias was not detected through statistical testing, it is hard to completely rule it out since the funnel plot seems to be asymmetrical. Finally, the predominance of retrospective and moderate‑quality studies also introduces potential biases, such as selection bias, which may affect the validity of our findings.

Future research should prioritize prospective, multicenter studies with standardized definitions and diagnostic criteria for PVT. This would enhance comparability of the data and provide a more accurate estimate of PVT prevalence. Moreover, clinical trials are needed to evaluate the efficacy and safety of prophylactic anticoagulation in high‑risk populations, such as patients with liver cirrhosis or those undergoing extensive vascular resection. The role of postoperative anatomical changes, particularly portal vein angle, in PVT development also warrants further investigation. Understanding the impact of surgical modifications on vascular dynamics may offer new strategies to prevent thrombosis. Finally, the relationship between intraoperative factors (eg, blood transfusion) and PVT remains an area for further study, particularly in the context of optimizing transfusion protocols to balance the risks of bleeding and thrombosis.

## CONCLUSIONS

PVT remains a significant complication following hepatectomy, particularly in the patients with liver cirrhosis, those undergoing PVR, and those with notable postoperative anatomical changes. Identifying these risk factors allows for targeted perioperative interventions that may reduce the incidence of PVT and improve overall patient outcomes. Future research should aim to validate these findings through well‑designed prospective studies and to explore preventive measures, including individualized anticoagulation strategies, to further enhance postoperative care for high‑risk patients.
